# Supplementary material for: Risk of Cervical Carcinoma After Unfavorable Behavior and High Genetic Risk in the UK Biobank: A Prospective Nested Case–Control Study
Source: Biomedicines. 2025 Feb 13;13(2):464. doi: 10.3390/biomedicines13020464 (PMC11853234; doi:10.3390/biomedicines13020464)
Supplement: Supplementary file 1 [file biomedicines-13-00464-s001.zip › biomedicines-3415546-supplementary.pdf]

**Supplementary Text 1**

**Sexual & reproductive behavior related questions used in the current analysis**

| Questions | Response | Hints |
|---|---|---|
| How many children have you given birth to? | Enter INTEGER<br>OR<br>-3: Prefer not to answer<br>-1: Do not know | Please include live births only.<br>If you are unsure, please provide an estimate or select Do not know. |
| What was your age when you first had sexual intercourse? (Includes vaginal, oral or anal intercourse) | Enter INTEGER<br>OR<br>-2: Never had sex<br>-3: Prefer not to answer<br>-1: Do not know | If you are unsure, please provide an estimate or select Do not know. |
| About how many sexual partners have you had in your lifetime? | Enter INTEGER<br>OR<br>-3: Prefer not to answer<br>-1: Do not know | If you are unsure, please provide an estimate or select Do not know. |

## UK Biobank data fields used in this study

| Data field ID | Description |
|---|---|
| 53 | Date of attending assessment centre |
| 33 | Date of birth |
| 40000 | Date of death |
| 191 | Date lost to follow-up |
| 40005 | Date of cancer diagnosis |
| 2734 | Number of live births |
| 2139 | Age first had sexual intercourse |
| 2149 | Lifetime number of sexual partners |
| 2784 | Ever taken oral contraceptive pill |
| 21001 | BMI |
| 20116 | Smoking status |

## Supplementary Text 2

### Polygenic risk score

**UK Biobank genotyping and quality control.** We used the imputed genotypes from UKB. Details of the design of the array, sample processing and quality control have been described in detail elsewhere. From the resulting dataset, we extracted a European ancestry subset (408,812 individuals), including samples who self-identified as white British and had very similar genetic ancestry based on a principal component analysis of the genotypes. The variants with minor allele frequency (MAF) <0.001, Hardy–Weinberg equilibrium test $P$ value $<1.0\times10^{-12}$, missing genotype rate >0.05, or imputation accuracy score <0.3 were excluded by using PLINK.

**Polygenic risk score construction for cervical carcinoma**. We constructed the cervical carcinoma PRS for individuals in UKB based on the GWAS summary statistics in the FinnGen study (data freeze 8, Fall 2021), which included 2,913 cases and 149,394 controls. The clumping and threshold approach was applied to construct the cervical carcinoma PRS by selecting the significant ($P<5\times10^{-8}$) and independent SNPs ($r^2<0.1$) in 1,000 kb region based on genotypes of 1,000 randomly selected European samples from UKB. The effect sizes of 21 significant and independent SNPs were derived from the FinnGen study (Table S1). We summed the effect of all selected SNPs together into a PRS as the following model,

$$PRS_i=\sum\beta_k X_{ik}$$

where $\beta_k$ was the effect size of the variant k in the FinnGen study, and $X_{ik}$ was the number of effective alleles for SNP k ($X_{ik}$=0, 1, or 2) of i$^{th}$ participant in UKB.

## Supplementary Text 3

## Power calculations

Assuming at least 310 individuals with cervical carcinoma, 60% of controls had age at first intercourse <= 18 years old, and a case-control ratio of 1:20, the study has 80.27% power to detect an odds ratio of at-least 1.41 or greater with a type I error of 2.5%. Under the assumption that 65% of the control group had several sexual partners greater than or equal to two, the study has 99.61% power to detect an odds ratio of at-least 1.80 or greater. In a hypothetical 20% of controls with live births greater than 2, the study has 85.09% power to detect an odds ratio of at-least 1.50 or greater.

**Supplementary Text 4**

**Association of polygenic risk score with cervical carcinoma.** We constructed the PRS for cervical carcinoma in the UK Biobank with the weight of 21 independent SNPs from the FinnGen study (Table S1). Across the whole population, the cervical carcinoma cases (n= 5,262), including the incident and prevalent cases, had higher PRS compared to the controls (n= 192,658) with $P < 0.001$ using t-test (Figure S1A). The proportion of cervical carcinoma cases tended to increase with higher PRS, ranging from 1.65 per hundred in the first decile to 3.77 per hundred in the last decile (Figure S1B). Compared to the individuals with PRS in the first decile, those in the highest decile had over two times the risk with an OR of 2.33 (Figure S1C).

**Table S1: SNPs associated with cervical carcinoma susceptibility.**

| SNP | CHR | BP | Ref | Alt | EAF | Beta | Se | P | N |
|---|---|---|---|---|---|---|---|---|---|
| rs62404144 | 6 | 32634990 | G | T | 0.0872489 | -0.55771 | 0.0537168 | 2.982E-25 | 152307 |
| rs9271929 | 6 | 32596650 | A | C | 0.50086 | -0.233982 | 0.0263522 | 6.7437E-19 | 152307 |
| rs114786106 | 6 | 32596878 | A | G | 0.122745 | -0.383098 | 0.0439267 | 2.7504E-18 | 152307 |
| rs2856820 | 6 | 33046083 | C | T | 0.340897 | -0.245243 | 0.0284763 | 7.168E-18 | 152307 |
| rs57165260 | 6 | 31836202 | A | C | 0.199008 | -0.273993 | 0.0348625 | 3.8646E-15 | 152307 |
| rs11751024 | 6 | 32586236 | C | A | 0.397819 | -0.198695 | 0.0272378 | 2.9902E-13 | 152307 |
| rs149772142 | 6 | 32386395 | T | C | 0.0296384 | -0.659593 | 0.0945526 | 3.0381E-12 | 152307 |
| rs1053726 | 6 | 31322047 | A | G | 0.212027 | -0.218866 | 0.0337219 | 8.5664E-11 | 152307 |
| rs9266228 | 6 | 31325320 | G | C | 0.608218 | -0.172248 | 0.0267305 | 1.1648E-10 | 152307 |
| rs12603332 | 17 | 38082807 | T | C | 0.46226 | -0.168007 | 0.0266321 | 2.8184E-10 | 152307 |
| rs113742050 | 6 | 32575432 | C | T | 0.0181254 | 0.530003 | 0.0841825 | 3.0568E-10 | 152307 |
| rs113617605 | 6 | 31478752 | G | C | 0.0220672 | -0.696577 | 0.110698 | 3.1217E-10 | 152307 |
| rs206018 | 6 | 32177880 | C | G | 0.245951 | -0.197814 | 0.0316317 | 4.0089E-10 | 152307 |
| rs31484 | 5 | 1337906 | A | T | 0.481635 | -0.162198 | 0.0264486 | 8.6459E-10 | 152307 |
| rs35580488 | 6 | 32097421 | G | C | 0.0198102 | -0.722815 | 0.118036 | 9.1435E-10 | 152307 |
| rs115806028 | 6 | 33200889 | G | C | 0.0995947 | -0.290984 | 0.0477278 | 1.0825E-09 | 152307 |
| rs6457711 | 6 | 33045272 | C | A | 0.302093 | -0.177177 | 0.029484 | 1.8638E-09 | 152307 |
| rs9269109 | 6 | 32443266 | T | C | 0.679806 | -0.165822 | 0.027701 | 2.149E-09 | 152307 |
| rs9277552 | 6 | 33055501 | C | T | 0.165688 | 0.196636 | 0.0339185 | 6.7388E-09 | 152307 |
| rs12660769 | 6 | 32272310 | C | G | 0.213443 | -0.192173 | 0.0334904 | 9.5706E-09 | 152307 |
| rs62399431 | 6 | 30993880 | G | C | 0.240492 | -0.174534 | 0.0319412 | 4.6497E-08 | 152307 |

**Table S2: Association between cervical carcinoma and behavior score and genetic risk based on weighted score.**

| Characteristics | | Adjusted OR[a] (95% CI) | P | Adjusted OR[b] (95% CI) | P |
|---|---|---|---|---|---|
| Behavior Score | Favorable | 1 | | 1 | |
| | Intermediate | 2.64 (1.89 - 3.69) | <0.0001 | 2.63 (1.88 - 3.68) | <0.0001 |
| | Unfavorable | 4.12 (2.66 - 6.37) | <0.0001 | 4.00 (2.58 - 6.2) | <0.0001 |
| Genetic risk category | Low | | | 1 | |
| | Intermediate | | | 1.24 (0.93 - 1.65) | 0.15 |
| | High | | | 1.61 (1.17 - 2.2) | 0.0032 |

[a]: Calculated using multivariable conditional logistic regression model, adjusted for BMI and oral contraceptive use.

[b]: Calculated using multivariable conditional logistic regression model, adjusted for BMI, oral contraceptive use, PRS, the first ten principal components of genetic ancestry and genotyping batch.

**Table S3: Risk of cervical carcinoma according to genetic and behavior profiles based on weighted scores.**

| Genetic risk category | Behavior Score | | |
|---|---|---|---|
| | Favorable | Intermediate | Unfavorable |
| Low | 1 | 3.37 (1.5 - 7.54); P=0.0031 | 6.86 (2.61 - 18.01); P<0.0001 |
| Intermediate | 1.61 (0.68 - 3.8); P=0.28 | 4.37 (2.01 - 9.5); P=0.00020 | 5.46 (2.27 - 13.14); P=0.00015 |
| High | 2.38 (0.98 - 5.8); P=0.056 | 5.27 (2.39 - 11.63); P<0.0001 | 8.90 (3.47 - 22.79); P<0.0001 |

Calculated using multivariable conditional logistic regression model, adjusted for the first ten principal components of genetic ancestry, genotyping batch, BMI and oral contraceptive use.

**Table S4: Multivariable conditional logistic regression of cervical carcinoma risk based on matching ratio 1:10.**

| Characteristics | | Model 1[a] | | Model 2[b] | |
|---|---|---|---|---|---|
| | | Adjusted OR[c] (95% CI) | P | Adjusted OR[c] (95% CI) | P |
| Behavior Score | Favorable | 1 | | 1 | |
| | Intermediate | 1.65 (1.25 - 2.17) | 0.0005 | 2.58 (1.83 - 3.64) | <0.0001 |
| | Unfavorable | 2.45 (1.73 - 3.48 | <0.0001 | 4.14 (2.62 - 6.52) | <0.0001 |
| Genetic risk category | Low | 1 | | 1 | |
| | Intermediate | 1.24 (0.92 - 1.67) | 0.16 | 1.26 (0.94 - 1.70) | 0.12 |
| | High | 1.59 (1.15 - 2.20) | 0.0054 | 1.60 (1.15 - 2.21) | 0.0050 |

[a]: Scoring based on the presence or absence of risk factors. Behavior scores were classified as “favorable” (0 or 1 risk factor), “intermediate” (2 risk factors), or “unfavorable” (3 risk factors).

[b]: Scoring based on regression coefficients. Behavior scores were classified as “favorable” (Score 0-2), “intermediate” (Score 3-5), or “unfavorable” (Score 6).

[c]: Calculated using multivariable conditional logistic regression model, adjusted for BMI, oral contraceptive use, PRS, the first ten principal components of genetic ancestry and genotyping batch.

**Table S5: Multivariable conditional logistic regression of cervical carcinoma risk based on matching ratio 1:40.**

| Characteristics | | Model 1[a] | | Model 2[b] | |
|---|---|---|---|---|---|
| | | Adjusted OR[c] (95% CI) | P | Adjusted OR[c] (95% CI) | P |
| Behavior Score | Favorable | 1 | | 1 | |
| | Intermediate | 1.74 (1.33 - 2.28) | <0.0001 | 2.66 (1.94 - 3.63) | <0.0001 |
| | Unfavorable | 2.69 (1.93 - 3.75) | <0.0001 | 3.74 (2.41 - 5.81) | <0.0001 |
| Genetic risk category | Low | 1 | | 1 | |
| | Intermediate | 1.23 (0.92 - 1.64) | 0.16 | 1.23 (0.93 - 1.64) | 0.15 |
| | High | 1.54 (1.13 - 2.11) | 0.0065 | 1.55 (1.14 - 2.12) | 0.0058 |

[a]: Scoring based on the presence or absence of risk factors. Behavior scores were classified as “favorable” (0 or 1 risk factor), “intermediate” (2 risk factors), or “unfavorable” (3 risk factors).

[b]: Scoring based on regression coefficients. Behavior scores were classified as “favorable” (Score 0-2), “intermediate” (Score 3-5), or “unfavorable” (Score 6).

[c]: Calculated using multivariable conditional logistic regression model, adjusted for BMI, oral contraceptive use, PRS, the first ten principal components of genetic ancestry and genotyping batch.

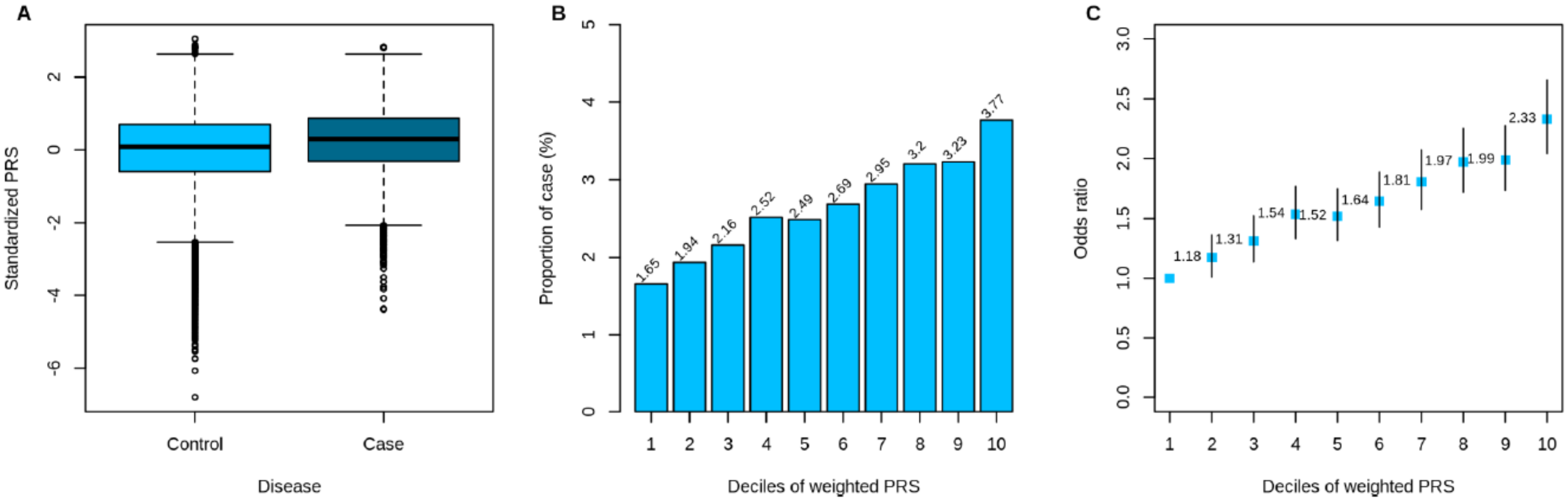


**Figure S1: Risk for cervical cancer according to PRS.** (A) PRS distribution among cervical cancer cases versus controls in the UK Biobank. Within each boxplot, the horizontal lines reflect the median, the top and bottom of each box reflect the interquartile range within each group. (B) The proportion of cervical cancer cases in ten groups according to the deciles of PRS. (C) The odds ratio of each group compared with those in the first decile group. Error bars are 95% confidence intervals.

A

| Genetic risk | Behavior score | Adjusted ORs (95% CIs) | P |
|---|---|---|---|
| Low | Favorable | ref | |
| | Intermediate | 1.36 (0.75-2.49) | 0.31 |
| | Unfavorable | 2.28 (1.21-4.31) | 0.011 |
| Intermediate | Favorable | 1.90 (1.01-3.55) | 0.045 |
| | Intermediate | 2.60 (1.47-4.59) | 0.0011 |
| | Unfavorable | 2.79 (1.52-5.11) | 0.0009 |
| High | Favorable | 3.82 (1.80-8.14) | 0.0005 |
| | Intermediate | 3.23 (1.68-6.21) | 0.0005 |
| | Unfavorable | 4.50 (2.20-9.22) | <0.0001 |

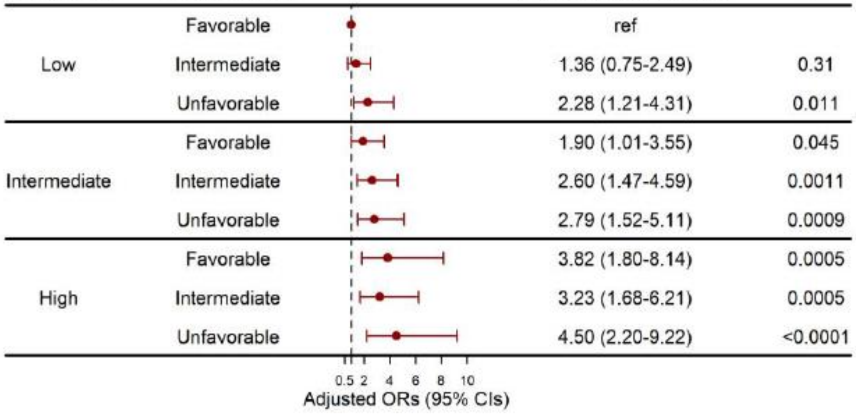


B

| Genetic risk | Behavior score | Adjusted ORs (95% CIs) | P |
|---|---|---|---|
| Low | Favorable | ref | |
| | Intermediate | 1.36 (0.57-3.22) | 0.48 |
| | Unfavorable | 2.60 (1.06-6.42) | 0.038 |
| Intermediate | Favorable | 3.05 (1.35-6.90) | 0.0074 |
| | Intermediate | 4.24 (1.94-9.29) | 0.0003 |
| | Unfavorable | 4.63 (2.08-10.32) | 0.0002 |
| High | Favorable | 6.76 (2.55-17.90) | 0.0001 |
| | Intermediate | 5.10 (2.07-12.54) | 0.0004 |
| | Unfavorable | 9.22 (3.49-24.38) | <0.0001 |

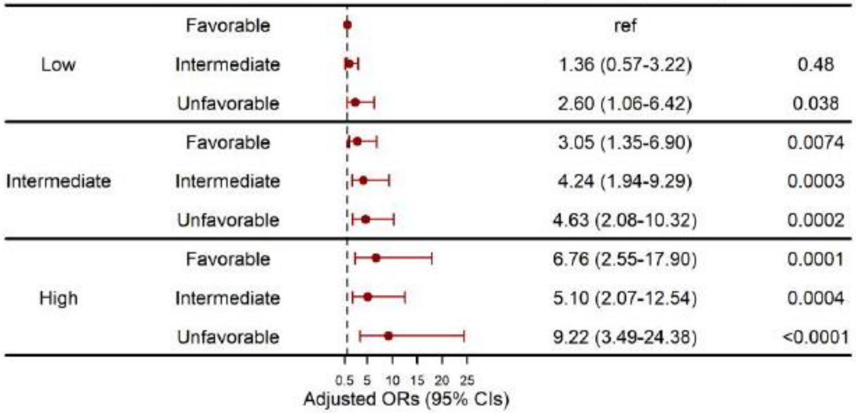

**Figure S2: Joint effects of the behavior score and PRS on the risk of cervical carcinoma based on the matching ratio of 1:10.** (A) Model based on the number of risk factors. (B) Model based on the regression coefficients

A

| Genetic risk | Behavior score | Adjusted ORs (95% CIs) | P |
|---|---|---|---|
| Low | Favorable | ref | |
| | Intermediate | 1.47 (0.82-2.65) | 0.20 |
| | Unfavorable | 2.26 (1.22-4.17) | 0.0096 |
| Intermediate | Favorable | 2.30 (1.25-4.23) | 0.0073 |
| | Intermediate | 2.74 (1.57-4.79) | 0.0004 |
| | Unfavorable | 2.97 (1.65-5.36) | 0.0003 |
| High | Favorable | 3.61 (1.74-7.50) | 0.0006 |
| | Intermediate | 3.89 (2.07-7.29) | <0.0001 |
| | Unfavorable | 5.28 (2.66-10.47) | <0.0001 |

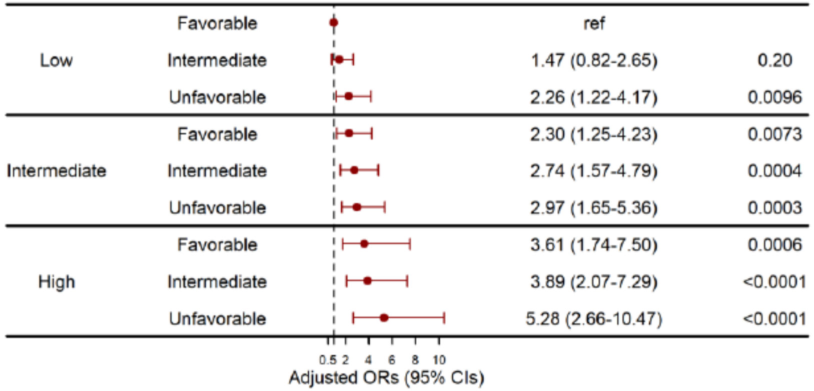


B

| Genetic risk | Behavior score | Adjusted ORs (95% CIs) | P |
|---|---|---|---|
| Low | Favorable | ref | |
| | Intermediate | 1.40 (0.68-2.89) | 0.36 |
| | Unfavorable | 1.70 (0.77-3.73) | 0.19 |
| Intermediate | Favorable | 2.80 (1.40-5.61) | 0.0037 |
| | Intermediate | 3.64 (1.89-7.02) | 0.0001 |
| | Unfavorable | 4.53 (2.31-8.90) | <0.0001 |
| High | Favorable | 5.56 (2.31-13.39) | 0.0001 |
| | Intermediate | 4.33 (1.93-9.72) | 0.0004 |
| | Unfavorable | 6.26 (2.59-15.11) | <0.0001 |

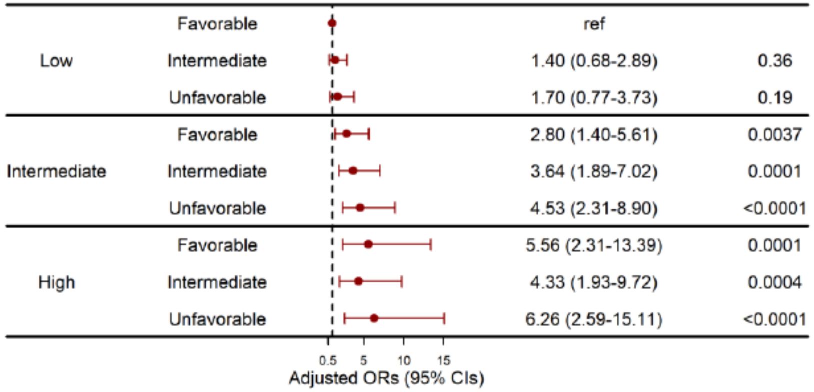

**Figure S3: Joint effects of the behavior score and PRS on the risk of cervical carcinoma based on the matching ratio of 1:40.** (A) Model based on the number of risk factors. (B) Model based on the regression coefficients.
